# Effect of an expansion in private sector provision of contraceptive supplies on horizontal inequity in modern contraceptive use: evidence from Africa and Asia

**DOI:** 10.1186/1475-9276-10-33

**Published:** 2011-08-19

**Authors:** David R Hotchkiss, Deepali Godha, Mai Do

**Affiliations:** 1 Department of Global Health Systems and Development, School of Public Health and Tropical Medicine, Tulane University, New Orleans, USA

## Abstract

**Background:**

One strategic approach available to policy makers to improve the availability of reproductive and child health care supplies and services as well as the sustainability of programs is to expand the role of the private sector in providing these services. However, critics of this approach argue that increased reliance on the private sector will not serve the needs of the poor, and could lead to increases in socio-economic disparities in the use of health care services. The purpose of this study is to investigate whether the expansion of the role of private providers in the provision of modern contraceptive supplies is associated with increased horizontal inequity in modern contraceptive use.

**Methods:**

The study is based on multiple rounds of Demographic and Health Survey data from four selected countries (Nigeria, Uganda, Bangladesh, and Indonesia) in which there was an increase in the private sector supply of contraceptives. The methodology involves estimating concentration indices to assess the degree of inequality and inequity in contraceptive use by wealth groups across time. In order to measure inequity in the use of modern contraceptives, the study uses multivariate methods to control for differences in the need for family planning services in relation to household wealth.

**Results:**

The results suggest that the expansion of the private commercial sector supply of contraceptives in the four study countries did not lead to increased inequity in the use of modern contraceptives. In Nigeria and Uganda, inequity actually decreased over time; while in Bangladesh and Indonesia, inequity fluctuated.

**Conclusions:**

The study results do not offer support to the hypothesis that the increased role of the private commercial sector in the supply of contraceptive supplies led to increased inequity in modern contraceptive use.

## Background

One strategic approach available to policy makers to improve the availability of reproductive and child health care services in low- and middle-income countries is to expand the role of the private sector in providing these services. There are a number of arguments that are used to support this type of strategy. First, the private sector may be more efficient than the public sector in the provision of services to those households who are willing and able to pay, particularly those who live in urban areas. Secondly, a strategy that involves working with the private sector can help mobilize additional resources for reproductive and child health programs. Third, increasing the private sector's market share can potentially allow population and health programs to better target the poor and other vulnerable households who have limited physical and financial access to services. However, critics of this approach argue that increased reliance on the private sector will not serve the needs of the poor, and could lead to increases in socioeconomic disparities in the use of services.

Family planning services is one example of a reproductive and child health care service where there has been much attention on the role of the private sector. Over the past ten years, the demand for family planning services has increased dramatically, as evidenced by large increases in modern contraceptive prevalence rates (MCPR) and growing numbers of women entering childbearing ages in many low- and middle-income countries [1]. However, during the same period, donor financing for family planning programs has diminished and, in some countries, been phased out [2]. Taken together, both trends can potentially threaten the continuation of current levels of MCPR as well as progress towards the long-term sustainability of family planning programs. In response, many countries have turned to the private sector for the provision of contraceptive supplies and services. This may be due to a shortfall of public resources for the health sector, poor governance, and a deliberate strategy to engage the private sector [3].

There is little research available that investigates the relationship between the expansion of the private sector in the provision of contraceptive supplies and socioeconomic disparities in modern contraceptive use. One exception is a recent study by Agha and Do [4], which employed population-based survey data from five countries-Morocco, Indonesia, Kenya, Ghana, and Bangladesh. The authors found no support for the hypothesis that an increase in the private sector supply of family planning services leads to socioeconomic inequality in the MCPR.

In this study, we revisit the question of whether the expansion of the role of private providers in selected countries in Africa and Asia has led to increased socio-economic disparities in modern contraceptive method use. The countries included in the analysis are Uganda, Nigeria, Bangladesh, and Indonesia, all of which have experienced an increase in the share of women using the private commercial sector for their contraceptive supplies.

The study methods build on those of Agha and Do [4]. Like that study, we use multiple rounds of Demographic and Health Survey (DHS) data for selected countries where there was an increase in the private sector supply of contraceptives to estimate concentration indices, whch are used to assess the degree of inequality in contraceptive use by wealth groups, across time. However, we extend their analysis by also investigating whether the expansion of the role of private providers is associated with increased horizontal inequity in modern contraceptive use.

We define inequality as differences in contraceptive use between wealth groups. Inequality is different from inequity, which we define as unequal use for equal need (horizontal inequity), the standard definition used in the health equity literature [5]. In our case, inequality is unequal contraceptive use between wealth groups, regardless of the need for family planning, while inequity is unequal contraceptive use for equal need for family planning. For example, if women in richer households are more likely to use a modern contraception method than women in poorer households, then the inequality does not necessarily mean that there is inequity because the variation in contraceptive use between wealth groups might be explained by socioeconomic variation in the need for family planning. In order to measure the extent of MCPR inequity in each of the study countries, the study controls for differences in the need for family planning (FP) services in relation to household wealth. This allows us to measure the extent of horizontal inequity in contraceptive use. The analysis is based on DHS data from Uganda, Nigeria, Bangladesh, and Indonesia. In the latter two countries, also analyzed by Agha and Do [4], we incorporate into our analysis of trends data from a more recent DHS round.

This paper is organized as follows. After this introductory section, section 2 describes the data and methods used in the study. Section 3 presents study the empirical results of our analysis. Finally, section 4 presents a discussion of the results and the policy implications for family planning decision-makers interested in improving the availability of FP services as well as the sustainability of FP programs.

## Methods

### Data sources

This study utilizes data from DHS, which are nationally representative population-based surveys of women of reproductive age (15 to 49 years of age). The use of standardized questionnaires in the DHS makes it possible to examine changes in the variables of interest across multiple countries. For each country included in the study, the final sample consists of women of reproductive age who are either currently married or living in union.

### Inclusion criteria

For the purposes of this study, countries were initially selected if: a) there were at least three rounds of DHS available; and b) there was an expansion in the private commercial sector as source of supply for modern contraceptives in three consecutive surveys. The initial search for countries that met our criteria was conducted using STAT COMPILER, which includes data from all DHS [6]. This was followed by accessing each of the available DHS data sets for countries that were identified and then eliminating those countries where the private commercial sector share did not expand, using the study's definition of the private commercial sector (which does not include nongovernmental organizations [NGOs]). After applying these criteria, the following seven countries remained: Nigeria, Uganda, Namibia, Zimbabwe, Morocco, Indonesia, and Bangladesh. Due to the budget constraint of the study, we selected four of these countries: Nigeria, Uganda, Bangladesh, and Indonesia. Of the four, two countries were not included in the analysis by Agha and Do [4]. For the two countries also included in Agha and Do [4], Bangladesh and Indonesia, more recent DHS had been conducted and made available for each country, which provide an opportunity to test the robustness of their results. Table 1 in the appendix lists the surveys used for the four study countries and their respective sample sizes.

**Table 1:** Surveys Used in the Analysis

Country	Year of DHS survey	Sample size (currently married or cohabitating women)
**Nigeria**	2008	23,954
	2003	5,157
	1999	5,755
		
**Uganda**	2006	5,362
	2001	4,675
	1995	4,903
	1988	3,055
		
**Bangladesh**	2007	10,146
	2004	10,417
	1999-00	9,530
	1996-97	8,306
	1993-94	8,846
		
**Indonesia**	2007	30,869
	2003	27,784
	1997	26,833
	1994	26,220
	1991	21,187
	1987	10,919

### Variables

The variable of primary interest in the study is current modern contraceptive use, a binary variable derived from the responses to the question, "Are you currently doing something or using any method to delay or avoid getting pregnant?" and, for those women who answered yes, "Which method are you using?". The methods classified as modern are male condoms, pills, intrauterine device (IUD), injections, diaphragm/foam/jelly, female sterilization, male sterilization, and Norplant. Lactational amenorrhea method (LAM) was not classified as a modern method.

Also of interest is an indicator of whether the woman received her contraceptive supplies from a private commercial provider. This indicator is based on the response to the question asked to women who were using a contraceptive method, "Where did you obtain [current method] the last time?" For the purposes of this study, we define the private commercial sector as consisting of those commercial outlets that sell contraceptive supplies and services, including chemists, shops, pharmacies, traditional healer/doctor, midwife, and private health care facilities and workers^1^. This excludes NGOs and faith-based organizations (FBOs). Based on this definition, we generated an indicator of the source of supply with three categories: the private commercial sector, the government sector, and other sources (NGOs, relatives, friends, and others). We then used this variable to assess changes over time in the extent to which women received FP supplies from a private commercial sector outlet "the last time" the method was obtained^2^.

In order to control for need in the equity analysis, a variable on the need for family planning services was generated from the responses to questions on the desire for more children at the time of the survey. A woman was classified as having a need for family planning if she: 1) wanted a child no sooner than two years following the survey, 2) wanted a child but was unsure of the timing, 3) was undecided on whether she wanted more children, 4) did not want more children, 5) was sterilized at the time of the survey, 6) was currently pregnant at the time of the survey but had wanted the current pregnancy later or not at all, or 7) was postpartum amenorrhic and who had wanted the last birth later or not at all. Like the commonly used measure of unmet need, we classified women who wanted a child within the next two years and women who were "infecund" (barren) as not being in need of contraception. Furthermore, all contraceptive users who had missing information on the "desire for more children" were also classified as women in need^3^. Note that the indicator of need does not consider whether the woman is using a contraceptive method, which makes our definition different than that used in the DHS.

In order to assess variation in the use of modern contraception by socioeconomic status, a composite measure of household wealth was generated based on questions on household assets and living conditions using principal components analysis, which was then used to rank and assign households to wealth quintiles, along the lines suggested by Filmer and Pritchett [7].

### Analytical approach

To quantify socioeconomic inequality in modern contraceptive use in the analysis, a concentration index (CI) was calculated for each survey round. The values of the CI can range from -1.0 to +1.0, with 0 indicating no inequality, a negative value indicating increased concentration of modern contraceptive use among the poor, and a positive value indicating increased concentration among the rich.

A potential problem with the CI approach above is that it does not consider differences in women's need for family planning services by socioeconomic status, and therefore limits the extent to which one can measure inequities in modern contraceptive use, as opposed to inequalities. In order to investigate horizontal inequity^4 ^in modern contraceptive use in each of the surveys, we standardized the measure of modern contraceptive use for family planning need in relation to household wealth. This was done using the indirect method of standardization, as suggested by the World Bank Institute [8].

The following steps were carried out to assess horizontal inequity. First, need-predicted modern contraceptive use is estimated using probit regression models. The dependent variable in the models is a dichotomous indicator measuring whether the woman is currently using a contraceptive method. Two types of independent variables were included in the models. The first type is composed of "need variables" measuring the need for modern contraception. Need variables in this study consist of the dichotomous indicator of need described above, as well as the age and the educational attainment of the woman. The second type is composed of "non-need" variables, which are correlates of utilization of modern contraception that may bias the coefficients of the need variables if omitted from the models^5 ^[8]. The non-need variables, which are non-confounding variables as they are theoretically related only to modern contraceptive use and not to family planning need, consist of a household wealth score, the partner's educational attainment, woman's employment status, and region (urban vs. rural).

Second, the results of the model are used to estimate the woman's need-predicted probability of modern contraceptive use by setting the non-need variables at their means, and then generating predicted values.

Third, need-standardized modern contraceptive use is obtained by adding the overall sample mean of the indicator of modern contraceptive use to the difference between actual and need-predicted modern contraceptive use.

Fourth, once need-expected and need-standardized use were obtained, we calculate their respective concentration indices. The method of indirect standardization "corrects" the actual distribution by comparing it with the distribution that would be observed if all women had not their levels of the non-need variables but the same mean values of the non-need variables as the entire population [8]. The CI of need-standardized contraceptive use provides a measure of horizontal equity.

A key assumption of this analysis is that once observable need indicators have been controlled, "any residual variation in utilization is attributable to non-need factors" [8]. This may be a strong assumption, given that the variables used to measure need were based on information on the desire for more children, age, and educational attainment. If there is unobserved variation in need correlated with wealth, then the procedure will result in biased measurement of horizontal inequity. Unfortunately, the modeling approach used in the study does not allow us to test this assumption with our data.

## Results

This section presents the empirical results for the four study countries. We first describe changes in the share of who report relying on the private commerical sector for their contraceptive supplies and in the modern contraceptive prevelance rate over time. We then present changes in the values of the concentration indices both for actual and need-standardized MCPR. Finally, to help explain changes in MCPR inequity, we explore changes in the extent to which poor women relied on the private commercial sector for their contraceptive supplies. Comparisons between countries should be made with caution due to variation in the policy, economic, political, and cultural context as well as the length of the study period. An overview of the context in each of the study countries can be found in Hotchkiss et al. [9].

### Share of all women relying on the private sector

**Figure 1:**
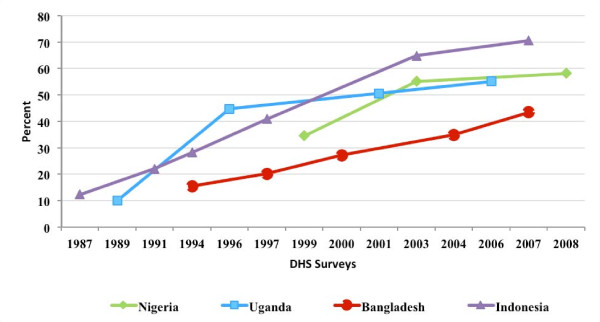
**Percent of women who report relying on the private commercial sector for their contraceptive supplies, by country and by year**.

Figure 1 presents the study results on the changes in the share of women who report obtaining the contraceptive supplies from the commercial private sector. As mentioned in the methods section, a country must have experienced an increase in the role of the private sector to be eligible for the study. In each of the four study countries, the increase in the private commercial share was substantial, ranging from 69 percent over the 1999 to 2008 period in Nigeria to 476 percent over the 1987 to 2007 period in Indonesia. The explanation for why the private commercial share increased in the study countries is not entirely clear. In Nigeria and Uganda, and Indonesia the increase may have been the result of both an explicit programmatic strategy to socially market contraceptive supplies, as well as a fluctuating public sector support for family planning due to political and macro-economic forces [10-17]. In Bangladesh, the increase may have been the result of the government's strategy to increase the role of private sector [18,19].

### Modern contraceptive prevalance rate

**Figure 2:**
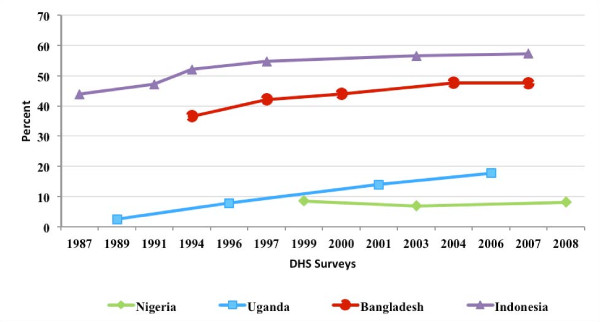
**Current use of modern contraceptive methods, by country and by year**.

Figure 2 presents the MCPR over time in each of the study countries. As the private commercial share of contraceptives increased, the MCPR increased in Uganda, Bangladesh, and Indonesia, and stagnated in Nigeria. Changes in the mix of methods used by the sample women was not investigated in the study.

### MCPR inequality and inequity

**Figure 3:**
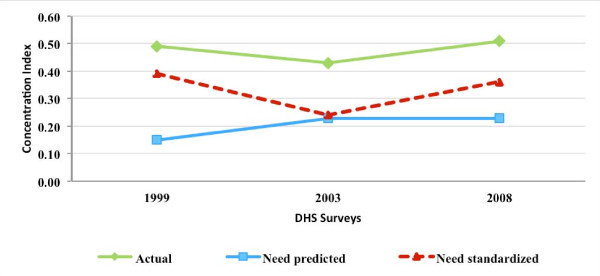
**Concentration indices for actual, need-predicted and need-standardized current use of modern contraceptives-Nigeria**.

**Figure 4:**
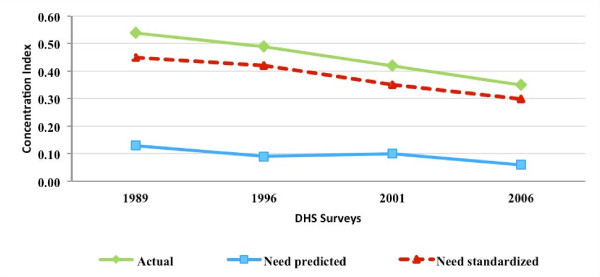
**Concentration indices for actual, need-predicted and need-standardized current use of modern contraceptives-Uganda**.

**Figure 5:**
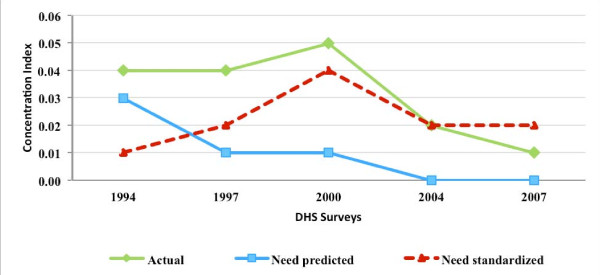
**Concentration indices for actual, need-predicted and need-standardized current use of modern contraceptives-Bangladesh**.

**Figure 6:**
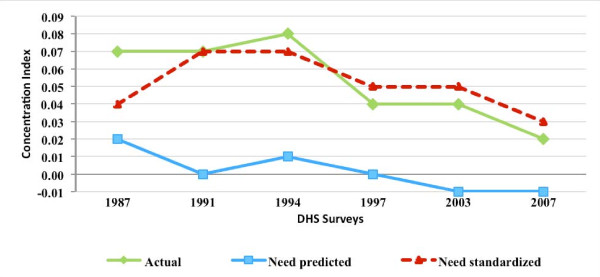
**Concentration indices for actual, need-predicted and need-standardized current use of modern contraceptives-Indonesia**.

Figures 3, 4, 5, and 6 present for Nigeria, Uganda, Bangladesh, and Indonesia, respectively, the estimated concentration indices for actual, need-predicted, and need-standardized contraceptive use. As described in the methods section, the indicator of MCPR inequality is the concentration index for actual modern contraceptive use. The study results suggest that in Nigeria and Uganda, actual modern contraceptive use was concentrated among the rich during the study period, with the CI relatively stable in Nigeria from 1999 to 2008 but declining in Uganda from 1988 to 2006. In the two Asian countries, Bangladesh and Indonesia, actual modern contraceptive use was only slightly pro rich, with the CI declining from 0.04 in 1994 to 0.01 in 2007 in Bangladesh and declining from 0.07 in 1987 to 0.02 in 2007 in Indonesia.

In Nigeria and Uganda, the results for need-standardized CI, which measures MCPR inequity as opposed to MCPR inequality, shows a lower pro-rich distribution in modern contraceptive use than the actual distribution. This is indicated by the CI for the need-standardized distribution being lower than that of the actual distribution in each of the three survey years. For example, in 1999, the CI for the need-standardized distribution was 0.39, compared to the CI of 0.49 for the actual distribution (please see Table 2 in the appendix for 95 percent confidence intervals for each of the concentration indices estimated). It should be noted that, in each of the survey years in both Nigeria and Uganda, MCPR inequity was relatively high while the MCPR among poor women was quite low (results not shown).

**Table 2:** Estimates of Concentration Indices and 95% Confidence Intervals, by Survey

DHS Survey	Type of Concentration Index	Concentration Index	(95% Confidence Interval)
Nigeria 1999	Actual CI	0.49	(0.48-0.50)
	Need predicted CI	0.15	(0.14-0.16)
	Need standardized CI	0.39	(0.35-0.42)
Nigeria 2003	Actual CI	0.43	(0.41-0.44)
	Need predicted CI	0.23	(0.21-0.25)
	Need standardized CI	0.24	(0.17-0.31)
Nigeria 2008	Actual CI	0.51	(0.51-0.52)
	Need predicted CI	0.23	(0.23-0.24)
	Need standardized CI	0.36	(0.33-0.39)
Uganda 1988	Actual CI	0.54	(0.42-0.67)
	Need predicted CI	0.13	(0.10-0.15)
	Need standardized CI	0.45	(0.33-0.57)
Uganda 1995	Actual CI	0.49	(0.45-0.53)
	Need predicted CI	0.09	(0.07-0.10)
	Need standardized CI	0.42	(0.38-0.47)
Uganda 2001	Actual CI	0.42	(0.39-0.45)
	Need predicted CI	0.10	(0.09-0.11)
	Need standardized CI	0.35	(0.31-0.38)
Uganda 2006	Actual CI	0.35	(0.31-0.39)
	Need predicted CI	0.06	(0.05-0.06)
	Need standardized CI	0.30	(0.26-0.34)
Bangladesh 1993-94	Actual CI	0.04	(0.02-0.05)
	Need predicted CI	0.03	(0.02-0.03)
	Need standardized CI	0.01	((-0.01)-0.03)
Bangladesh 1996-97	Actual CI	0.04	(0.02-0.05)
	Need predicted CI	0.01	(0.01-0.02)
	Need standardized CI	0.02	(0.01-0.04)
Bangladesh 1999-00	Actual CI	0.05	(0.04-0.06)
	Need predicted CI	0.01	(0.01-0.02)
	Need standardized CI	0.04	(0.02-0.05)
Bangladesh 2004	Actual CI	0.02	(0.00-0.03)
	Need predicted CI	0.00	(-0.00-0.01)
	Need standardized CI	0.02	(0.01-0.02)
Bangladesh 2007	Actual CI	0.01	(0.01-0.02)
	Need predicted CI	0.00	((-0.01)-0.00)
	Need standardized CI	0.02	(0.01-0.02)
Indonesia 1987	Actual CI	0.07	(0.05-0.08)
	Need predicted CI	0.02	(0.02-0.03)
	Need standardized CI	0.04	(0.03-0.06)
Indonesia 1991	Actual CI	0.07	(0.06-0.08)
	Need predicted CI	0.00	(0.00-0.01)
	Need standardized CI	0.07	(0.05-0.08)
Indonesia 1994	Actual CI	0.08	(0.07-0.09)
	Need predicted CI	0.01	(0.00-0.01)
	Need standardized CI	0.07	(0.06-0.08)
Indonesia 1997	Actual CI	0.04	(0.03-0.05)
	Need predicted CI	0.00	((-0.01)-0.00)
	Need standardized CI	0.05	(0.04-0.05)
Indonesia 2003	Actual CI	0.04	(0.03-0.05)
	Need predicted CI	-0.01	((-0.02)-(-0.01))
	Need standardized CI	0.05	(0.04-0.06)
Indonesia 2007	Actual CI	0.02	(0.02-0.03)
	Need predicted CI	-0.01	((-0.01)-(-0.01))
	Need standardized CI	0.03	(0.03-0.04)

In contrast to the two sub-Saharan African countries, there was relative little difference between the actual and need-standardized distributions in Bangladesh and Indonesia. This is due to the need-expected probability of modern contraceptive use among currently married women being relatively uniform across the five wealth groups in each of the survey years. Overall, the level of MCPR inequity, based on the need-standardized distribution, remained relatively constant during a time when the private commercial sector was expanding in Bangladesh and Indonesia.

### Share of poor women relying on the private sector

**Figure 7:**
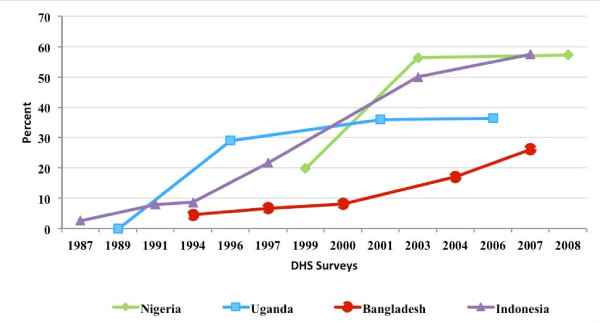
**Percent of poor women who report relying on the private commercial sector for their contraceptive supplies, by country and by year**.

Figure 7 presents the share of contracepive users in the poorest wealth quintile who report relying on the private commercial sector over time. As can be seen, in each of the study countries, poor women became increasingly reliant on the private commercial sector over time. Moreover, in Nigeria and Indonesia, the private commercial sector became the most important source of contraceptive supplies to women in poorest wealth quintile group. In addition to the the poorest quintile, women in better off wealth quinties also became increasingly reliant on the private commericial sector in each of the four study countries.

## Discussion

The purpose of this study is to investigate whether the expansion of the private commercial sector in the provision of contraceptive supplies leads to MCPR inequity. By facilitating the expansion of the role of the private sector, governments can potentially better target those women who are in need of family planning services, but lack the ability and willingness to pay. This can improve the likelihood that family planning programs will be financially sustainable, and help withstand fluctuations in donor assistance earmarked for family planning services. On the other hand, one could argue that if countries increasingly rely on the private sector without appropriate adjustment of the targeting of services to the poor and other vulnerable groups, the availability of contraceptives to those groups could potentially deteriorate, and as a result, lead to MCPR inequality (and inequity). Because the relationship between increased private market share and MCPR inequity is not obvious, empirical evidence on this issue is needed by reproductive health policy makers in low- and middle-income countries who are responsible for improving contraceptive security.

Overall, the results of the study suggest that the expansion of the private commercial sector supply of contraceptives in the two African study countries (Nigeria and Uganda) and the two Asian study countries (Bangladesh and Indonesia) did not lead to increased MCPR inequity. In fact, in Nigeria and Uganda, MCPR inequity actually decreased over time, while in Bangladesh and Indonesia, MCPR inequity, which was already quite low, fluctuated.

There are a number of important contextual differences between the four study countries that make it difficult to make definitive policy recommendations based on the results of the study.

First, in some of the countries, the expansion of the private commercial sector was not always part of an explicit government strategy. For example, the increased reliance of women on the private commercial sector for their contraceptive supplies was in part due to political and economic instability (i.e., Nigeria during the 1990s, Indonesia during the late 1990's and early 2000's, where the public sector's role diminished significantly) and in part due to family planning receiving lower priority in the population and health sectors (i.e., Nigeria during the 1990s, Uganda during 1990s, and Indonesia during the 2000s). This indicates that the private commercial sector helped fill a void that resulted from these macro-level forces. On the other hand, in Bangladesh, the expansion of the private sector seemed to be part of a deliberate policy strategy that shifted from a target-driven approach to a facility-based approach.

Second, the role of socially marketed contraceptives, which are included in our definition of the private commercial sector, may have also varied across the study countries. While social marketing played an important role in the family planning program in all four of the study countries, we do not have information on the degree to which the social marketing programs received price subsidies as well as the reach of the programs.

Third, while countries that increasingly relied on the private commercial sector for their family planning supplies should have had a greater ability to target public subsidies to poor women, the study results suggest that poor women's reliance on the public sector for their supplies did not increase over time. On the contrary, in each of the four study countries, women in the poorest wealth quintile increased their reliance on the private commercial sector while achieving higher rates of modern contraceptive use over time^6^. These results imply that the private commercial sector can play an important role in improving the availability and use of family planning supplies not only among better off women, but among poorer women as well.

In exploring the relationship between the expansion of the private commercial sector and MCPR inequity, a contribution of the study is that we control for the need for family planning services, which could potentially vary by socio-economic status and as a result, lead to differences between MCPR inequality, which is based on actual use, and MCPR inequity, which is based on need-standardized use. We control for need by deriving need-expected probabilities of using modern contraceptives, which are then used to calculate need-standardized concentration indices. We find that there are often substantial differences between the actual and need-standardized probabilities of modern contraceptive use, and as a result, the degree of MCPR inequity and the degree of MCPR inequality. This is particularly true in the two African countries included in our study.

There are a number of limitations to the study. First, we do not attempt to empirically attribute differences in MCPR inequity over time to differences in the private commercial supply. The family planning supply environment is one of many factors that can influence a woman's choice of where to obtain family planning supplies and services, along with other community-level factors and household- and individual-level factors. Second, the study is at the national level, and as such, may mask increases in MCPR inequity that may have occurred in some regions and districts of the study countries. Third, the study does not investigate whether the increased role of the private sector influences access to and use of long acting and permanent methods (LAPM) and short-term methods. Other limitations of the study include the relatively small number of women currently using contraception in Nigeria and Uganda, which may make it difficult to interpret changes over time, and the inclusion of socially marketed product provision in the definition of the private sector, which may have resulted in an overestimate of the size of the private commercial sector and, as a result, an underestimate of the true association between the private commercial sector's share of contraceptive supply and inequity.

## Conclusions

Our findings that the expansion of the private commercial sector did not lead to increased MCPR inequity in the four study countries are consistent with the conclusions of Agha and Do [4]. While the public sector remains an important source of supply for poor women, who may lack the physical and financial accessibility to private outlets that sell modern contraceptives, our results also suggest that the private commercial sector can also be an important source of supply to poor women without leading to increased MCPR inequity. Social marketing programs are likely to have played an important role in expanding the use of private suppliers among poor women.

## Competing interests

The authors declare that they have no competing interests.

## Authors' contributions

The study was conceived by DRH, DG, and MD, designed and undertaken by DRH, DG, and MD, and written by DRH and DG. All the authors have read and approved the final manuscript.

## End-notes

^1 ^It is possible that private workers may be public workers who are moonlighting, but our data does not allow us to investigate the importance of moonlighting in the study countries.

^2 ^For Indonesia, the PPKBD (village family planning posts), posyandus (health posts), and polindes (delivery posts) have been classified as public facilities in the 1987 and 1991 DHS but as 'other private' sources in 1994, 1997, 2003 and 2007 DHS. A similar classification was used in this study with these facilities being classified as 'public' sources in 1987 and 1991 survey data and as 'NGO and other' sources for all other surveys.

^3 ^The number of missing cases is, for the most part, small. Each survey used but one had seven or fewer missing cases. The one survey used that has more than seven missing cases is the 1999 Nigeria DHS, which has 30 missing cases.

^4 ^Horizontal equity is defined as equal contraceptive use for women with equal need for contraceptives.

^5 ^This provides partial correlation of the standardizing variable with the variable of interest conditional on the presence of the non-confounding variables.

^6 ^In Bangladesh, the public sector share among the poorest fluctuated a bit but increased from 1994 to 2007. This is the only country where the public sector remains the main supplier for the poor while the private sector is increasingly the main provider for the rich.
